# Early Life Social Stress Causes Sex- and Region-Dependent Dopaminergic Changes that Are Prevented by Minocycline

**DOI:** 10.1007/s12035-022-02830-6

**Published:** 2022-04-18

**Authors:** Clarissa Catale, Luisa Lo Iacono, Alessandro Martini, Constantin Heil, Ezia Guatteo, Nicola Biagio Mercuri, Maria Teresa Viscomi, Daniela Palacios, Valeria Carola

**Affiliations:** 1 Division of Experimental Neuroscience, Neurobiology of Behavior Laboratory, IRCCS Santa Lucia Foundation, Rome, Italy; 2 Department of Dynamic and Clinical Psychology, and Health Studies, Sapienza University of Rome, Via degli Apuli 1, Rome, Italy; 3 Division of Experimental Neuroscience, Experimental Neurology Laboratory, IRCCS Santa Lucia Foundation, Rome, Italy; 4 Division of Experimental Neuroscience, Epigenetics and Signal Transduction Laboratory, IRCCS Santa Lucia Foundation, Rome, Italy; 5 Department of Motor Science and Wellness, University of Naples Parthenope, Naples, Italy; 6 Department of Systems Medicine, Università Degli Studi Di Roma Tor Vergata, Rome, Italy; 7 Department of Life Science and Public Health, Section of Histology and Embryology, Università Cattolica Del S. Cuore, Rome, Italy; 8 IRCCS Fondazione Policlinico Universitario A. Gemelli, Rome, Italy; 9 Department of Life Science and Public Health, Section of Biology, Università Cattolica Del S. Cuore, Rome, Italy

**Keywords:** Early life stress, Sex differences, Ventral tegmental area, Substantia nigra, Microglia, RNA sequencing

## Abstract

Early life stress (ELS) is known to modify trajectories of brain dopaminergic development, but the mechanisms underlying have not been determined. ELS perturbs immune system and microglia reactivity, and inflammation and microglia influence dopaminergic transmission and development. Whether microglia mediate the effects of ELS on dopamine (DA) system development is still unknown. We explored the effects of repeated early social stress on development of the dopaminergic system in male and female mice through histological, electrophysiological, and transcriptomic analyses. Furthermore, we tested whether these effects could be mediated by ELS-induced altered microglia/immune activity through a pharmacological approach. We found that social stress in early life altered DA neurons morphology, reduced dopamine transporter (DAT) and tyrosine hydroxylase expression, and lowered DAT-mediated currents in the ventral tegmental area but not substantia nigra of male mice only. Notably, stress-induced DA alterations were prevented by minocycline, an inhibitor of microglia activation. Transcriptome analysis in the developing male ventral tegmental area revealed that ELS caused downregulation of dopaminergic transmission and alteration in hormonal and peptide signaling pathways. Results from this study offer new insight into the mechanisms of stress response and altered brain dopaminergic maturation after ELS, providing evidence of neuroimmune interaction, sex differences, and regional specificity.

**Supplementary Information:**

The online version contains supplementary material available at 10.1007/s12035-022-02830-6.

## Introduction

Adverse childhood experiences can modify trajectories of brain development, leading to aberrant maturation that increases risk for psychopathologies and neurological disorders [1, 2]. Among the most consistent changes observed in maltreated individuals are alterations in structure and function of reward-associated brain regions, specifically the mesocorticolimbic and nigrostriatal dopaminergic pathways, which originate in the ventral tegmental area (VTA) and substantia nigra (SN), respectively [1, 3–5]. Such dopaminergic alterations have been associated with all kinds of mental disorders and are thought to be etiological factors for certain psychopathological outcomes, especially substance use disorders [6–9]. In this context, human research is still mostly correlational because of the difficulty in performing studies that reliably draw causal inference, especially in children [10]. Animal studies have demonstrated that early life stress (ELS) induces changes in the dopamine (DA) system in terms of excitability, receptor number, receptor sensitivity, DA reuptake, and metabolism as a function of the age of exposure to the stress, the life stage at which the stress effects are measured, and the type and duration of adversity [9, 11]. Moreover, these changes are paralleled by variations in behavioral domains that may predispose to psychiatric outcomes [4, 6, 9, 12–14]. Although preclinical research has contributed tremendously to this topic, knowledge about the biological mechanisms by which ELS alters development of the DA system is still scarce.

One of the actors involved in guiding DA system development is the brain’s resident immune compartment, namely microglia. Studies have established that during brain development, microglia contribute directly to the correct maturation of the DA system and DA-dependent behaviors, by regulating outgrowth of dopaminergic axons in the mouse embryonic forebrain [15] and pruning the levels of DA receptors in adolescent male rats [16]. Other studies have shown that ELS can trigger microglia “activation” in rodent models and an enduring systemic inflammatory phenotype in both humans and animals [17, 18]. Based on these findings, it has been proposed that ELS interferes with microglia and immune system developmental trajectories, resulting in aberrant neural maturation and brain function and increased risk for neurodevelopmental, neuropsychiatric, and neurological disorders [17–20]. However, whether and how ELS modifies the interaction between microglia and DA system during development is still unknown.

Recently, we reported that exposure to a threatening social environment (social stress, SS) in the juvenile period in mice brings about microglia activation and aberrant DA-induced self-inhibitory responses of DA neurons in the developing VTA, and sensitivity towards the effects of cocaine later in life [21, 22]. Treatment with minocycline, an inhibitor of microglia activation, or GW2580, a selective inhibitor of macroglia/macrophage functionality, during SS normalizes microglia and dopamine sensitivity in the VTA and prevents the development of cocaine susceptibility [22].

Following these results, here we used the SS model [20–25] to evaluate the impact of ELS on markers of dopaminergic functionality and development in the VTA and SN of male and female mice. Furthermore, we tested whether microglia were implicated in the emergence of SS-induced dopaminergic changes by using minocycline. Finally, a transcriptome analysis in control and SS pups VTA was performed to evaluate biological pathways involved in ELS-induced dopaminergic changes.

## Materials and Methods

### Animals and Breedings

Seven-week-old CD-1 (CD1) male and DBA2/J @Ico (DBA) male and female mice were purchased from Charles River Laboratories (Calco, Italy). DBA male and female mice were mated at 12 weeks of age, with max 3 females and 1 male per cage. Pregnant mothers were single housed around gestation day 16. Each male was used for producing max 2 litters, and each mother (*primipara*) was used for producing only one litter to rule out the effects of changes in maternal behavior across pregnancies. Mice were kept at constant temperature (21 ± 1 °C) and humidity (55 ± 5 percent). Food and water were provided ad libitum, and mice were housed on a 12:12 light:dark cycle with lights on at 07.00 a.m. All experiments were carried out in accordance with Italian national law (DL 26/2014) on the use of animals for research based on the European Communities Council Directive (2010/63/UE) and comply with the ARRIVE guidelines. Experiments were approved by the ethics committee of the Italian Ministry of Health (license/approval IDs: #42/2015-PR; #677/2019-PR) and by local Ethical Committee of the Santa Lucia Foundation. All efforts were made to minimize the number of animals used and their suffering.

### Early Life Social Stress

**Fig. 1:**
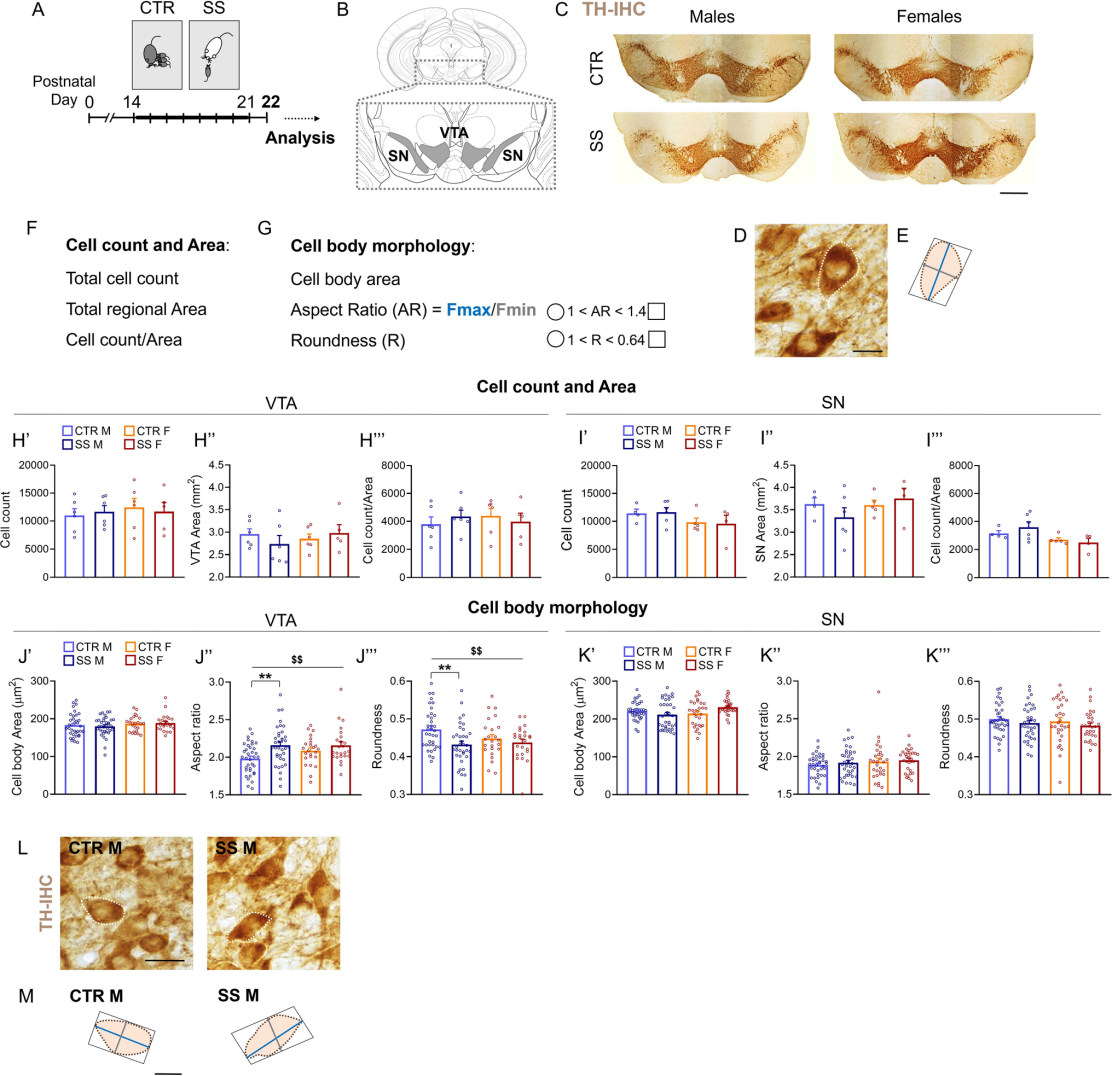
Early life social stress changes dopaminergic neurons morphology in the VTA but not SN of male mice only. **A** Social stress (SS) protocol was applied from postnatal days (PDs) 14 to 21 and animals were sacrificed at PD22. **B** Schematic of coronal ventral midbrain section containing the Ventral Tegmental Area (VTA) and Substantia Nigra (SN) (Bregma≈-3.52 mm, based on 27). Analyses were performed on the parabrachial pigmented and paranigral nuclei of the VTA and the pars compacta of the SN. **C**, **D** Representative images showing dopaminergic neurons in the VTA and SN at **C** lower and **D** higher magnification as revealed by tyrosine hydroxylase (TH) immunohistochemistry (IHC). **E** Representative reconstruction of a TH + neuron with maximum (Fmax, blue) and minimum (Fmin, gray) diameter (or feret) of the cell. **F**, **G** Parameters analyzed. **G** Aspect ratio (AR) is the ratio of Fmax and Fmin. A circle has an AR and R of 1 and a square has an AR of 1.4 and a R of 0.64. **H**’–**H**’’’, **I**’–**I**’’’ No significant difference in **H**’, **I**’ total TH + cell count, **H**’’, **I**’’ TH + area, and **H**’’’, **I**’’’ cell count/area was detected between SS and CTR mice in both regions and sexes. **J**’–**J**’’’ Significant differences in aspect ratio and roundness but not cell body area, were found between SS and CTR mice in the VTA (**J**’–**J**’’’; MANOVA, univariate group effect: *P* < 0.01). Specifically, SS significantly altered the shape of DA neurons somata in the VTA of male but not female mice, increasing the aspect ratio and decreasing the roundness (**J**’’, **J**’’’; Tukey HSD test: *P* < 0.001). Such differences were not detected in (**K**’–**K**’’’) male or female SN. Representative images (L) and reconstruction (M) of male VTA TH + neurons in CTR and SS mice. Dopaminergic neurons in SS mice appear more squared, with a longer maximum diameter (blue) and a shorter minimum diameter (gray). **H**’–**I**’’’ *N* = 5–6 animals/group/sex. Dots in the graphs represent average value for each animal. **J**’–**K**’’’ *N* = 4–6 animals/group/sex; 6 hemisections/animal. Dots in the graphs represent average value for each hemisection. Univariate group effect: $$ *P* < 0.01; Tukey HSD test: **P < 0.01. Data are presented as mean ± s.e.m. Scale bars = (C) 500 µm; (D, M) 10 µm, (L) 20 µm

The SS model is a validated model of early life social stress [21] established in our laboratory [20–25]. As previously demonstrated, the SS paradigm does not alter overall maternal behavior towards the pups [21]. DBA litters with an average number of 5 pups (min 4, max 6 pups) were included in the study. Mouse pup litters were randomly (blind to litter features) assigned to unhandled control (CTR) or SS group at postnatal day (PD) 14. In each litter, max 2 mice/sex/group were used for experiments shown here. In the CTR group, mothers and offspring were left undisturbed until sacrifice (PD22). In the SS group, each pup was housed in a cage with a resident adult CD1 male mouse for 30 min per day from PD 14 to 21 (Fig. 1A) [21]. To avoid physical attacks to the pups, CD1 males were gonadectomized and single housed 1 month before the beginning of early life stress procedures. Limited cage cleaning was performed in the offspring cage from birth to weaning to avoid handling of the pups. DBA mice were sacrificed at PD22 for experiments.

### Minocycline Treatment

For minocycline experiments, CTR and SS pups received daily intraperitoneal (i.p.) injection of minocycline hydrochloride (50 mg/kg; Sigma-Aldrich, #M9511) [22, 26] dissolved in sterile saline solution (2.5 mg/ml) from PD14 to PD21. For SS mice, injection was performed 1 h prior to the stress procedure [22].

### Immunohistochemistry

At PD22, 4–6 mice per sex per group were anesthetized with Rompun (20 mg/ml, 0.5 ml/kg, i.p., Bayer) and Zoletil (100 mg/ml, 0.5 ml/kg, Virbac) and perfused transcardially with 20-ml saline followed by 20 ml of 4% paraformaldehyde (PFA) in phosphate buffer (PB) (0.1 M, pH 7.4). Brains were post fixed in 4% PFA overnight at 4 °C and then immersed in a 30% sucrose-PB solution until sinking. Afterwards, brains were cut into 30-µm-thick coronal sections using a freezing microtome.

For stereological and morphological analyses of dopaminergic neurons, every second slice approximately between – 3.08 and – 3.80 mm from Bregma (based on [27]) containing the VTA and SN was selected, for a total of 8 sections. Sections were treated for 5 min with 0.3% H_2_O_2_ to block endogenous peroxidase and then incubated for 48 h at 4 °C with rabbit anti-tyrosine hydroxylase (TH) antibody (1:700; #sc-25269, Santa Cruz Biotechnologies) for detection of dopaminergic neurons. TH is the rate-limiting enzyme in the synthesis of dopamine and marks dopamine neurons in the VTA and SN. Then, sections were incubated for 2 h with a biotinylated donkey anti-rabbit antibody (1:200, #BA-1000, Vector Laboratories), followed by 2 h in an ExtrAvidin solution (1:1000; #E2886, Sigma-Aldrich) at room temperature. As chromogen, 3,3’diaminobenzidine 0.05% (#D5637, Sigma-Aldrich) was used. Primary and secondary antibody solutions as well as ExtrAvidin solutions were prepared in PB and 0.3% Triton X-100. Each incubation step was followed by three 5 min rinses in PB. The specificity of immunohistochemical labeling was evaluated based on established guidelines on TH + staining in the midbrain [28, 29]. Sections from all experimental groups were processed together to allow quantitative comparisons. Finally, sections were mounted on chrome-alum–coated slides, air-dried, dehydrated, and coverslipped.

**Fig. 2:**
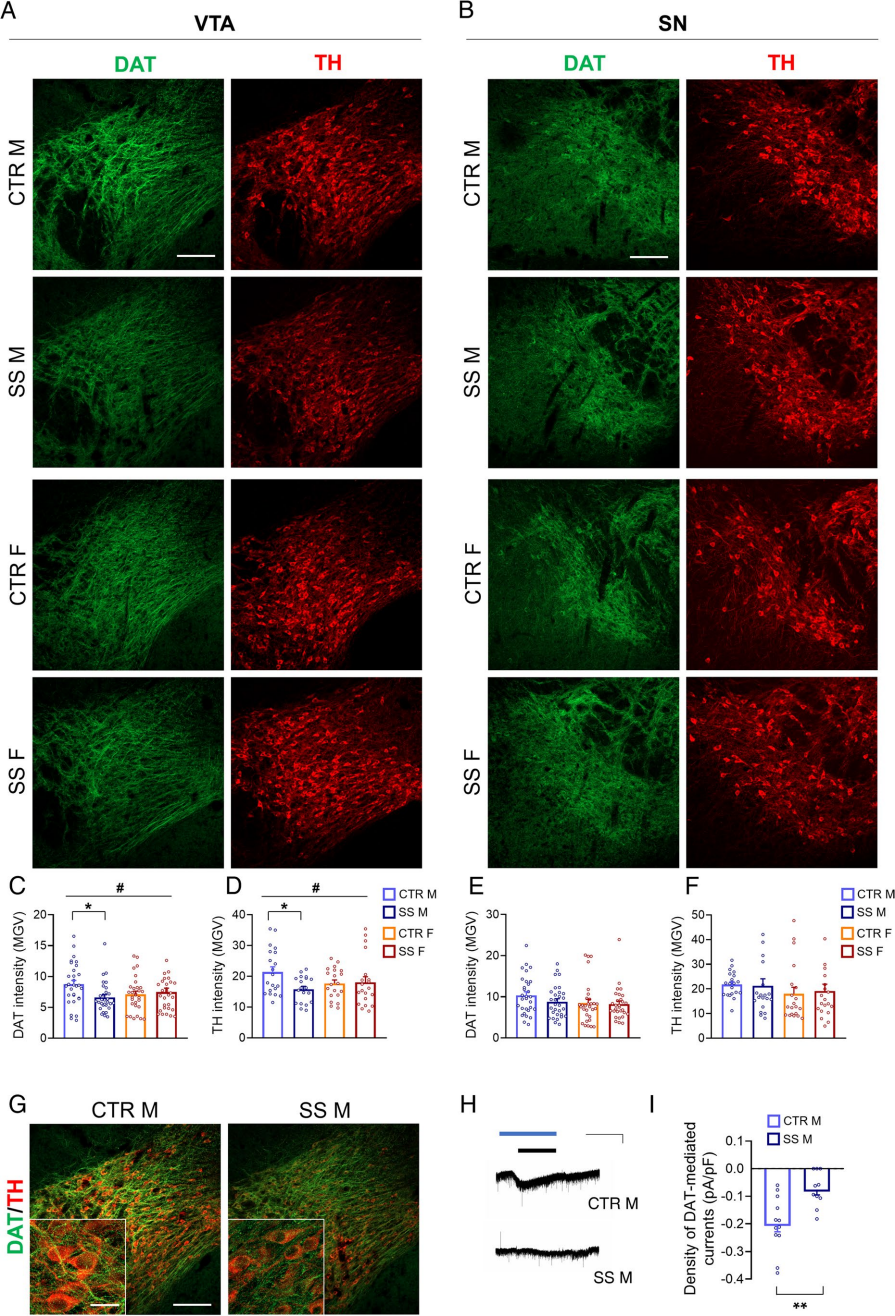
SS downregulates dopaminergic markers in the developing VTA but not SN of male mice only. **A**, **B**, **G** Representative coronal hemisection images and **C**–**F** quantification of DAT and TH expression in the VTA and SN of female and male PD22 pups from CTR and SS groups as revealed by immunofluorescence and confocal microscopy. **C**, **D** DAT and TH intensity (reported as mean gray value, MGV) was significantly reduced in the VTA of male SS mice with respect to CTR, but not in female mice (two-way ANOVA, group*treatment effect: *P* < 0.05; Tukey HSD test: *P* < 0.05). **E**, **F** No significant stress effect was detected in female and male SN. **G** Representative confocal images of merged DAT and TH signals in the male VTA showing lower immunoreactivity and sparse signal distribution in SS mice compared to CTR. **H** Representative traces of DAT-mediated inward currents induced by bath application of dopamine (DA) (black bar, 30 µM, 3 min) in the presence of sulpiride (blue bar, 5 µM, 5 min), a selective DRD2 antagonist, recorded by means of whole-cell patch clamp (Vh =  − 60 mV) from VTA DA neurons of CTR and SS midbrain slices (scale bar = 3 min, 30 pA). (I) Mean density of DA-induced, DAT-mediated currents, expressed as percentage of control currents, shows lower current amplitudes in SS compared with CTR neurons at PD22 (*t-*test, *P* < 0.01). **C**–**F** *N* = 5 animals/group/sex; 4–6 hemisections/animal. Dots in the graphs represent average value for each section. Group*treatment effect: # *P* < 0.05; Tukey HSD test: *P < 0.05. **I** *N* = 3 animals/group; 3–4 cells/animal. Dots in the graphs represent average value for each cell. Student’s *t*-test: **P < 0.01. Data are presented as mean ± s.e.m. Scale bars = (A, B, G) 100 µm; (G inset) 20 µm

For immunofluorescence staining, a total of 4–6 regularly spaced sections of VTA and SN (between – 3.08 and – 3.80 mm from Bregma, based on [27]) were selected for each animal. Sections were incubated for 48 h at 4 °C with the primary antibodies including rabbit anti-TH (1:700; #sc-25269, Santa Cruz Biotechnologies) and rat anti-DAT (1:200; #sc-32259, Santa Cruz Biotechnologies), followed by 2-h incubation of secondary antibodies including Alexa Fluor 555 conjugated donkey anti-rabbit (#A-31572, 1:200; ThermoFisher Scientific) and Alexa Fluor 488 conjugated donkey anti-rat (#A-21208) at room temperature. Primary and secondary antibody solutions were prepared in PB 0.3% Triton X-100, and each incubation step was followed by three 5-min rinses in PB. To avoid staining variability, sections from all groups were concomitantly incubated with the same cocktail of primary and secondary antibodies. The specificity of immunohistochemical labeling was confirmed by the omission of primary antibody and the use of normal serum instead (negative controls, Supplementary file1 Fig. 2). Sections were rinsed, DAPI-counterstained, mounted using an anti-fade medium (Fluoromount; Sigma-Aldrich), and coverslipped.

### Stereological and Morphological Analyses of Dopaminergic Neurons

Sections processed for TH immunohistochemistry were used for obtaining unbiased estimates of total number and morphology of TH + neurons, and total surface area of the VTA and SN. The boundaries of these areas were defined by TH staining, according to published guidelines [27]. In the VTA, analysis was restricted to the parabrachial pigmented nucleus (PBP) and paranigral nucleus (PN) subareas, where TH + neurons are known to express classical dopaminergic markers such as vesicular monoamine transporter 2, dopamine transporter (DAT), and the dopamine receptor D2 (DRD2), and project to regions implicated in reward processing, such as the nucleus accumbens [28, 29]. In the SN, analysis was performed in the pars compacta, where the majority of cell bodies are TH + . We applied an optical fractionator stereological design (bilateral count) using the Stereo Investigator System (MicroBrightField Europe e.K.) as previously described [30]. Data collection was done blind to the experimental group of each animal.

Neuronal morphology analysis on sections processed for TH immunohistochemistry was performed using an optical microscope (DMLB, Leica) equipped with a motorized stage and a camera connected to the Neurolucida 7.5 software (MicroBrightField) [31, 32]. Three sections containing the VTA/SN were selected, and 8 random cells in each hemisphere (6 hemisections including 3 right and 3 left VTAs and SNs) were included in the analysis, for a total of 48 cells per animal per structure. The VTA and SN were detected using the 5 × objective, and neuronal cells were analyzed with a 100 × oil-immersion objective. Cell bodies were manually outlined, and a set of parameters from the “Contour Measurement” analysis of the Neurolucida Software (https://www.mbfbioscience.com/help/si11/Content/Contours/contourmeasure.htm) were collected to evaluate somatic shape based on literature [31, 33]: cell body area, aspect ratio, and roundness. Aspect ratio is the ratio between feret maximum and feret minimum, and feret maximum and minimum are defined as “the largest and smallest dimensions of the contour as if a caliper was used to measure across the contour” (from Neurolucida User Guide, Fig. 1E). Only TH + neurons that displayed intact soma and round nuclei were included. For final figures, images were acquired with the 5 × or 100 × oil-immersion objectives, exported in TIFF format, and adjusted for contrast and brightness equally across the entire image and across groups. Representative images of ventral midbrain TH + areas (Fig. 1C) were reconstructed from single hemisphere images acquired with the 5 × objective.

### Quantitative Analysis of Fluorescence Images

Sections were examined through a confocal laser scanning microscope (Zeiss CLSM800). Acquisitions of one left or right hemisphere per section (hemisections) were performed by using a 20 × /0.50 objective (Plan-Apochromat, Zeiss) so that all samples within each experiment were captured using consistent settings for laser power and detection gain. Images were acquired at 1024 × 1024-pixel resolution (image size 638.9 μm per side) and 8-bit depth and exported in carl zeiss image format (.czi). PBP and PN subareas of the VTA, and distinction between VTA and SN were identified with the aid of an atlas [27]. Analyses were performed by using the ImageJ software [34] on .czi acquisitions. No changes in brightness and contrast were applied for the analyses. Background was removed by placing a small region of interest (ROI) (15 μm per side) on an area with no specific signal and applying the “BG (background) subtraction from ROI” plugin. Subsequently, TH and DAT signals were quantified by measuring fluorescence intensity as mean gray value within a ROI positioned on the PBP/PN (300 μm per side) and SN pars compacta (200 μm per side) in 4 (TH) or 6 (DAT) regularly spaced sections per animal. All analyses were performed blinded to the mouse experimental group. For final figures, images were acquired through the 20 × or oil immersion 63 × (insets) objectives, exported in TIFF format and contrast and brightness were adjusted equally across the entire image and across groups.

### Electrophysiological Experiment

Preparation of midbrain slices (*N* = 3 mice/group) was performed as described previously [22, 35]. Briefly, mice were anesthetized with halothane and decapitated. Horizontal midbrain slices (thickness 250 μm) containing the VTA were cut within artificial cerebrospinal fluid (aCSF, 22) at 6–8 °C and left to recover in a holding chamber (32–33 °C) for 30 min prior to usage. A single slice was placed into a recording chamber (0.6 mL) of an upright microscope (BX51WI; Olympus) and continuously perfused with aCSF (33 °C) saturated with a 95% O_2_/5% CO_2_ gas mixture at a constant flow rate (2.5 mL/min). Slices were visualized with a microscope’s in-build CCD camera (Photometrics Evolve). Referring to the mouse practical map of DA neuron location in distinct VTA subregions previously reported by our group [36] and others [37], we performed electrophysiological recordings from DA neurons located in the lateral and intermediate VTA. As opposed to medial VTA, DA neurons belonging to these subregions produce similar responses to exogenous dopamine application [36–38]. Cell-attached and whole-cell patch clamp recordings were conducted according to Lo Iacono et al. [22]. Only DA-sensitive neurons simultaneously meeting the following criteria were included: slow and regular pacemaker firing (0.5–4 Hz) in the cell-attached configuration, action potential duration ≥ 1.2 ms in the cell-attached configuration and Ih amplitude ≥  − 50 pA at − 120 mV in whole-cell configuration. Non-firing cells in the cell-attached configuration were abandoned.

DAT-mediated inward currents were elicited by bath application of DA in the presence of the DRD2 antagonist sulpiride, to prevent activation of large DRD2-GIRK2 inhibitory outward currents. Excitatory DAT-current is generated by uptake of DA (against concentration gradient) co-transported with 2 Na^+^ and 1 Cl^−^ along their ionic gradients [39–42], and reverts polarity at membrane potential more positive (− 47 mV) than holding potential (− 60 mV), thus causing a net excitation/inward current in DA neurons [43–45]. DA (30 µM; H8502, Sigma-Aldrich) and L-sulpiride (5 µM; Sigma-Aldrich) were bath applied via a three-way tap syringe containing the drug at final concentration, dissolved in warm aCSF, saturated with a 95% O_2_/5% CO_2_. To unmask the DA-induced inward current mediated by DAT [46], sulpiride was applied 5 min prior to DA application. Stock solutions were freshly prepared on the day of the experiment. Drugs were applied only once per slice, in order to avoid possible receptor desensitization and/or cross-interactions between cells, and within 10 min after the establishment of the whole-cell configuration. DA application lasted until the DA-induced inward current reached a plateau in the whole-cell configuration. All recordings were performed blinded to the experimental group.

### RNA Purification from VTA Punches

Male pups were decapitated at PD22 (*N* = 4/group) and brains were dissected, deprived of cerebellum and pons-medulla, and stored at − 80 °C. Punches of VTA were obtained from coronal brain slices no thicker than 300 μm according to Ventura et al. [47] and with the aid of a mouse brain atlas [27]. A stainless steel tube of 0.5 mm internal diameter was used to punch left and right VTAs, which were then collected in the same tube and stored in liquid nitrogen until further processing. RNA was then isolated using Total RNA purification Kit (Norgen Biotek). RNA from individual VTA samples (about 200 ng each) were quantified and quality tested by Agilent 2100 Bioanalyzer RNA assay (Agilent technologies). Only RNA samples with RIN value > 7.0 were included in the study.

### RNA Library Preparation and Sequencing

mRNA sequencing was performed at IGA technology service (Udine, Italy). Universal Plus mRNA-Seq kit (Tecan Genomics, Redwood City, CA) has been used for library preparation following the manufacturer’s instructions. Final libraries were checked with both Qubit 2.0 Fluorometer (Invitrogen, Carlsbad, CA) and Agilent Bioanalyzer DNA assay or Caliper (PerkinElmer, Waltham, MA). Libraries were then sequenced on single-end 75 bp mode on NextSeq 500 (Illumina, San Diego, CA). On average 30 million reads were produced per sample.

### Bioinformatic Analyses of RNA-seq Data

Read quality was asserted through FastQC, and quality/adapter trimming was carried out using Cutadapt. Single end reads were aligned to mouse reference mm10 using STAR aligner. Subsequently, reads were counted for genes using htseq-count (features from Ensemble gtf file, ftp://ftp.ensembl.org/pub/release-102/gtf/mus_musculus/). Read counts were then input into DESeq2 in R, and differential expression analysis was conducted with default options [48]. Genes were considered differentially expressed if the adjusted *p*-value was less than 0.05. Subsequent to scaling of raw read counts and execution of differential expression analysis, read counts were transformed by DESeq2’s variance stabilizing transformation (VST), in order to stabilize read count variance as a function of total reads number. Transformed gene expression for genes with adjusted *p*-value less than 0.05 was standardized and plotted in the heatmap. Rows and columns underwent hierarchical clustering, considering Euclidean distance as distance metric. Heatmap plot was performed using the pheatmap R-package. Gene ontology analyses and gene networks were obtained by Ingenuity pathway analysis (QIAGEN Redwood City, www.qiagen.com/ingenuity). Over-representation analysis was carried out using the R packages DOSE and ReactomePA [49, 50]. The significance of enrichment is determined through application of the hypergeometric test.

### RNA-seq Data Availability

RNA-Seq data are available through NCBI’s Gene Expression Omnibus (GEO) repository, under accession number GSE199136.

### Statistics

All data were first checked for normality by graphical inspection of the residuals’ distribution and for homogeneity of variances with the Levene’s test. VTA and SN were analyzed separately. Analysis of the main effects of group and sex, and interactions between these variables on immunohistochemical data was performed with two-way analysis of variance (ANOVA). In case of main factor or interaction significance (*P* < 0.05), ANOVA was followed by planned post hoc comparisons (Tukey HSD test) and nested (hierarchical) one-way ANOVA. Nested ANOVA was performed to take into account variability within samples (between observations from the same animal) as well as variability within and between groups [51]. In nested ANOVA, the group (CTR vs SS) was considered as fixed factor, and the animals and observations (cells/sections) were considered as random factors, and sample sizes (N of animals) in the groups and number of observations per sample were kept equal. Electrophysiological data were subjected to parametric Student’s *t*-test (*t*-test). Minocycline experiments were analyzed by two-way ANOVA, considering group (stress) and treatment (minocycline) as main factors. For all tests, significance was set at *P* < 0.05. All data are expressed as mean ± standard error of the mean (s.e.m.). Statistica software Version 12.0 (StatSoft, Tulsa, OK, USA) was used to perform the statistical analyses.

## Results

### Early Life Social Stress Does Not Alter the Number of Dopaminergic Neurons nor the Total Area of the VTA and SN

Since it has been demonstrated that dopaminergic neurons undergo a wave of physiological death/apoptosis at PD14 [52], and that social stress either in the first two postnatal weeks or in adulthood can affect the number of DA neurons [31, 53], we tested whether our model of social stress (SS) applied during the third postnatal week could alter the number of DA neurons and the total area of the VTA (PBP and PN) and SN (pars compacta) in male and female PD22 pups (Fig. 1A–C, F). DA neurons were identified by means of tyrosine hydroxylase (TH) immunostaining (Fig. 1C). Multivariate two-way ANOVA (MANOVA) of stereological measures (number of TH + neurons, total area of the TH + region, and number of cells/area) in the VTA failed to reveal any significant group (CTR vs SS; *λ* = 0.99, *F*_3,17_ = 0.07, *P* = 0.98), sex (M vs F; *λ* = 0.95, *F*_3,17_ = 0.31, *P* = 0.82), or group*sex (*λ* = 0.93, *F*_3,17_ = 0.44, *P* = 0.73) effect (Fig. 1H’-H’’’; males: *N* = 6 animals/group, females: *N* = 5–6/group). Similar results were obtained for the SN (Fig. 1I’-I’’’, MANOVA, group effect: *λ* = 0.92, *F*_3,13_ = 0.36, *P* = 0.78; sex effect: *λ* = 0.68, *F*_3,13_ = 2.03, *P* = 0.16; group*sex effect: *λ* = 0.87, *F*_3,13_ = 0.64, *P* = 0.60; males: *N* = 4–6 animals/group, females: *N* = 4–5/group). These results indicate that male and female CTR and SS mice display similar DA neurons number and VTA/SN total area.

### SS Alters the Morphology of Dopaminergic Neurons in the VTA but Not SN of Male Mice Only

Chronic adult social stress can reduce the size of DA neurons [53], and soma size and morphology of DA neurons have been associated with altered functionality of these cells and aberrant behaviors [12, 54]. Therefore, we characterized morphological profiles of the soma of DA neurons in SS and CTR mice by evaluating cell body area and other shape indices, including the aspect ratio and roundness [33] (Fig. 1D, E, G). MANOVA of somatic shape indices in the VTA showed a significant main effect of the group (*λ* = 0.93, *F*_3,114_ = 2.90; *P* = 0.036), but no significant effect of sex (*λ* = 0.97, *F*_3,114_ = 1.30; *P* = 0.29) or group*sex interaction (*λ* = 0.97, *F*_3,114_ = 1.30; *P* = 0.27; males: *N* = 6 animals/group, 6 hemisections/animal; females: *N* = 4 animals/group; 6 hemisections/animal). Univariate results indicated a significant group effect only on aspect ratio (*F*_1,116_ = 8.84; *P* = 0.0036) and roundness (*F*_1,116_ = 6.88; *P* = 0.0099), but not on cell body area (Fig. 1J’-J’’’). Post hoc comparison revealed significant differences between male CTR and SS mice (aspect ratio: *P* = 0.0060; roundness: *P* = 0.0071), but not between female CTR and SS animals (Fig. 1J’’,J’’’). The difference in somatic shape between male CTR and SS VTA was further confirmed by nested ANOVA (cell(animal(group)): aspect ratio *F*_1,564_ = 16.78, *P* < 0.001; roundness *F*_1,564_ = 20.43, *P* < 0.001; *N* = 6 animals/group, 48 cells/animal), demonstrating that SS male DA neurons present an altered, elongated shape, with reduced short axis and augmented long axis, resulting in increased aspect ratio and decreased roundness compared to CTR (Fig. 1L, M).

This effect was restricted to the VTA, as no significant main or interaction effect was detected in the SN (Fig. 1K’-K’’’, MANOVA group effect: *λ* = 0.97, *F*_3,126_ = 1.10, *P* = 0.35; sex effect: *λ* = 0.96, *F*_3,126_ = 1.70, *P* = 0.16; group*sex effect: *λ* = 0.95, *F*_3,126_ = 2.30, *P* = 0.077; males: *N* = 6 animals/group, 6 hemisections/animal; females: *N* = 5 animals/group; 6 hemisections/animal).

### SS Reduces Expression of the Dopamine Transporter and Tyrosine Hydroxylase Proteins in the Developing VTA but Not SN of Male Mice Only

We performed immunofluorescence staining and confocal imaging to assess distribution and expression of dopamine transporter (DAT) and tyrosine hydroxylase (TH) proteins (Fig. 2A, B), which are canonical markers of dopaminergic neurons [29, 55] and have shown to be modulated by adult and early life social stress [11]. Two-way ANOVA on quantitative measurements of DAT intensity showed significant group*sex interaction effect in the VTA (*F*_1,116_ = 5.07, *P* = 0.026) but not in the SN (*F*_1,116_ = 1.40, *P* = 0.24), and no main effect of group or sex in either region (Fig. 2C, E; *N* = 5 animals/group/sex, 6 sections/animal). Similar results were obtained for TH intensity (Fig. 2D, F, Two-way ANOVA, VTA group*sex effect: *F*_1,75_ = 4.29, *P* = 0.042; SN group*sex effect: *F*_1,76_ = 0.11, *P* = 0.75; *N* = 5 animals/group/sex, 4 sections/animal). Post hoc comparisons of VTA data revealed significant differences between male CTR and SS DAT (*P* = 0.039) and TH (*P* = 0.038) intensity, but not between female CTR and SS (Fig. 2C, D). This was further confirmed by nested ANOVA analysis of DAT and TH immunoreactivity in male CTR and SS VTA (section(animal(group)): DAT *F*_1,50_ = 12.48, *P* < 0.001; TH *F*_1,30_ = 18.26, *P* < 0.001), demonstrating that SS induced down-regulation of DAT and TH fluorescence intensity in male VTA compared to CTR.

### SS Reduces DAT Currents in the Developing Male VTA

To assess whether the DAT immunoreactivity reduction induced by SS in the male VTA was associated with changes in DAT-dependent currents, we performed electrophysiological recordings on individual DA neurons at PD22. We analyzed DA-induced DAT-mediated depolarizing currents in the lateral VTA. Using whole-cell voltage clamp recordings of individual DA neurons, we measured the amplitude of the inward current that developed in response to bath application of DA + sulpiride, a selective DRD2 antagonist, in midbrain slices. DA neurons of SS mice (*N* = 10 cells) displayed significant lower amplitudes of DAT dependent currents compared with CTR (*N* = 12 cells) (*t*-test: *t*_20_ =  − 3.41, *P* = 0.0028; *N* = 3–4 cells/animal, 3 animals/group; Fig. 2H, I).

### Minocycline Treatment During SS Restores Morphology of Dopaminergic Neurons and Expression of DAT and TH in SS Male Mice

**Fig. 3:**
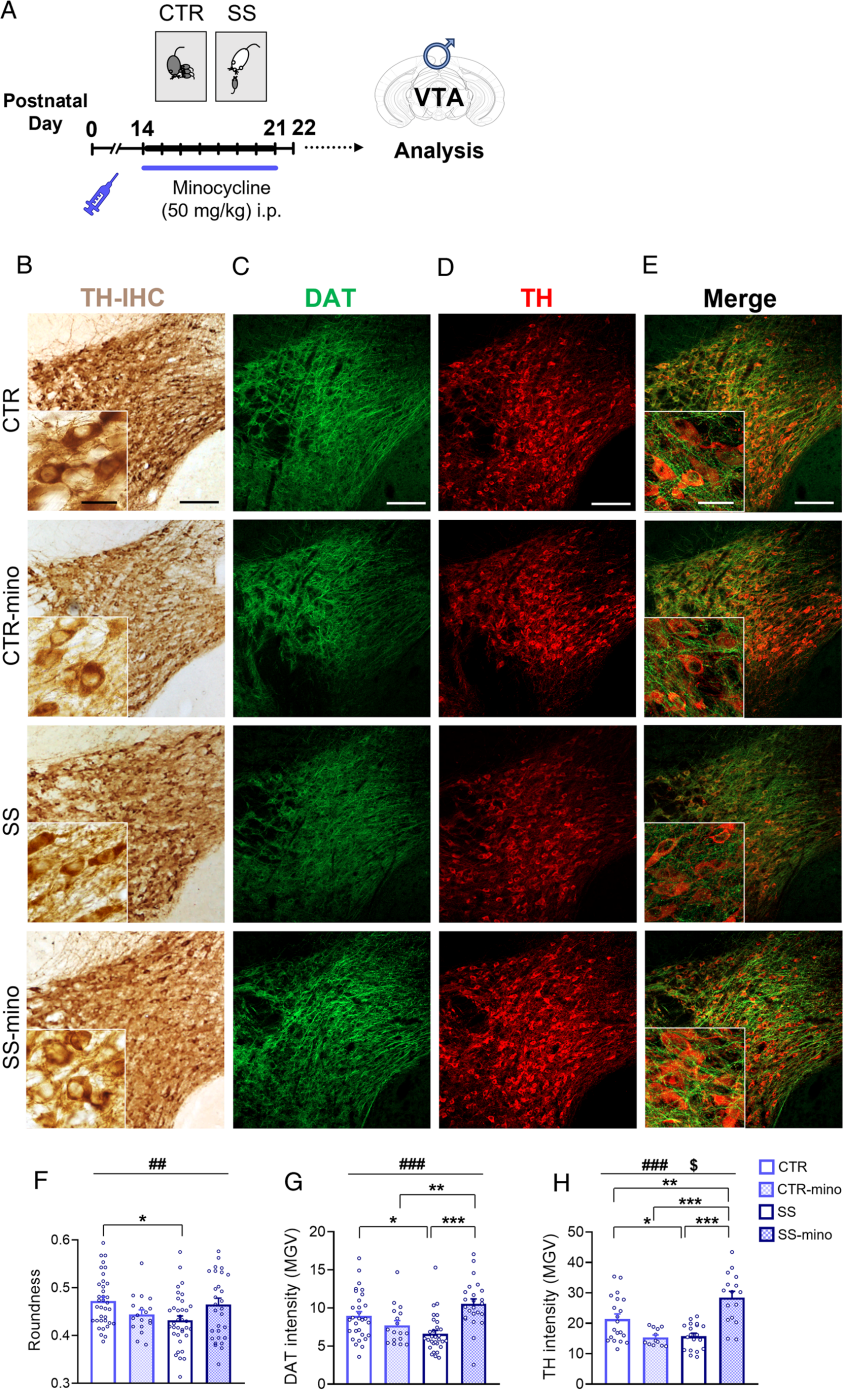
Minocycline treatment reverts dopaminergic aberrations induced by SS. **A** Minocycline was daily administered through intraperitoneal (i.p.) injection to male CTR and SS mice from PD14 to 21. Analyses were performed in the VTA at PD22. **B**–**E** Lower and higher (insets) magnification of **B** TH-IHC stained neurons, and **C**–**E** DAT and TH immunofluorescence in the VTA (coronal hemisection) of CTR, CTR-minocycline (CTR-mino), SS, and SS-minocycline (SS-mino) male pups. **F** Minocycline in SS mice restored roundness of dopaminergic neurons to CTR levels (MANOVA univariate group*treatment effect, *P* < 0.01). **G**, **H** In SS-mino mice, DAT, and TH immunoreactivity were significantly increased with respect to SS mice, to levels comparable to or above CTR mice. **F** *N* = 3–6 animals/group; 6 hemisections/animal. Dots in the graphs represent average value for each hemisection. Univariate group*treatment effect: ## *P* < 0.01; Tukey HSD test: *P < 0.05. **G**, **H** *N* = 3–5 animals/group; 4–6 sections/animal. Dots in the graphs represent value for each section. Group*treatment effect: ### *P* < 0.001; group effect: $ *P* < 0.05; Tukey HSD test: *P < 0.05, **P < 0.01, ***P < 0.001. Data are presented as mean ± s.e.m. Scale bars = (B-E) 100 µm, (B, E insets) 20 µm

We have previously demonstrated that SS induces microglia activation in the VTA [22]. Pharmacological inhibition of SS-induced microglia activation by systemic administration of minocycline prevented emergence of dopaminergic electrophysiological alterations at PD22 and cocaine conditioned place preference in adult stressed mice. Here, we employed minocycline to explore if and how microglia contributed to the observed morphological and molecular dopaminergic alterations caused by ELS exposure in the VTA of male mice (Fig. 3A).

First, to evaluate whether developmental minocycline treatment alone had an effect on dopaminergic markers and microglia cell number in CTRs, we compared TH intensity and ionized calcium-binding adapter molecule 1 (Iba1, microglia/macrophages marker) expression in CTR-saline and CTR-minocycline male VTA at PD22. TH intensity and Iba1 + cell count were comparable between minocycline- and saline-injected mice (TH: *t*-test: *t*_31_ = 1.42, *P* = 0.17; Iba1 + cell count: *t*-test: *t*_31_ = 1.58, *P* = 0.12; Supplementary file1 Fig. 1A-C), further supporting the specificity of minocycline action on microglia activation only in the SS condition [22].

Thus, we proceeded by comparing CTR, SS, CTR-minocycline, and SS-minocycline mice to evaluate group (stress) and treatment (minocycline) effects. MANOVA of somatic indices showed a significant effect of group (*λ* = 0.94; *F*_2,115_ = 3.90; *P* = 0.024), treatment (*λ* = 0.89; *F*_2,115_ = 7.10; *P* = 0.0011), and group*treatment interaction (*λ* = 0.89; *F*_2,115_ = 7.30; *P* < 0.001). Univariate results indicated a significant group*treatment effect only on roundness (*F*_1,116_ = 8.51; *P* = 0.0042), but not on the aspect ratio (*F*_1,116_ = 3.87; *P* = 0.052, data not shown), and no main effect of group or treatment on either index. Post hoc comparisons confirmed significant differences in the roundness only between CTR and SS untreated mice (*P* = 0.012), but no difference between the other groups, suggesting that roundness levels in SS-minocycline mice returned to CTR levels (Fig. 1B, F, CTR and SS: *N* = 6 animals/group, CTR-minocycline: *N* = 3 animals/group; SS-minocycline: *N* = 5 animals/group; all: 6 hemisections/animal).

Two-way ANOVA of DAT immunoreactivity showed a significant effect of group*treatment interaction (*F*_1,97_ = 19.95; *P* < 0.001), and no effect of the group or the treatment alone. Post hoc comparisons confirmed significant differences between CTR and SS untreated mice (*P* = 0.035), CTR-minocycline vs SS-minocycline (*P* = 0.0037), SS untreated vs SS-minocycline (*P* < 0.001), but no difference between CTR untreated and CTR-minocycline (*P* = 0.33), and in CTR untreated vs SS-minocycline (*P* = 0.13), indicating that minocycline treatment in SS mice restored immunoreactivity of DAT to CTR levels (Fig. 3C, G; CTR and SS: *N* = 5 animals/group, CTR-minocycline: *N* = 3 animals/group; SS-minocycline: *N* = 4 animals/group; 6 sections/animal).

Two-way ANOVA of TH immunoreactivity revealed significant main effect of group (*F*_1,63_ = 6.12, *P* = 0.016) and group*treatment interaction (*F*_1,63_ = 36.55, *P* < 0.001), but no effect of treatment alone. Post hoc comparisons showed significant differences between CTR and SS untreated mice (*P* = 0.035), CTR-minocycline vs SS-minocycline (*P* < 0.001), SS untreated vs SS-minocycline (*P* < 0.001), and CTR untreated vs SS-minocycline (*P* = 0.0084), indicating that minocycline treatment in SS mice (but not CTR mice) increased immunoreactivity of TH to levels above CTR (Fig. 3D, H; CTR and SS: *N* = 5 animals/group, CTR-minocycline: *N* = 3 animals/group; SS-minocycline: *N* = 4 animals/group; 4 sections/animal).

### SS Alters Transcriptomic Patterning in the Developing Male VTA

**Fig. 4:**
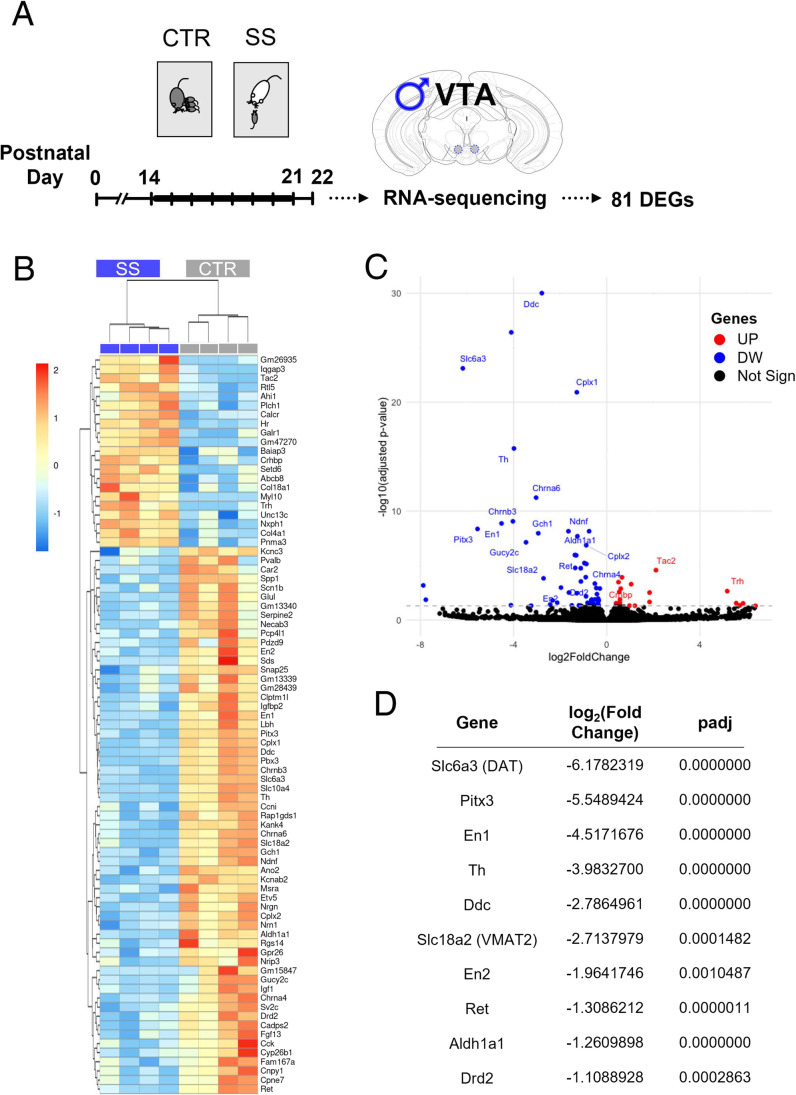
SS alters the transcriptome in the developing male VTA. **A** SS and CTR male VTA were collected at PD22 (*n* = 4/group). The drawing shows the location of the punches (bilateral 0.5 mm circular sampling). RNA-sequencing revealed 81 differentially expressed genes (DEGs) between the two groups. **B** Heatmap and **C** volcano plot of the DEGs. Downregulated genes in SS are highlighted in blue and upregulated genes are red (*p* adjusted < 0.05). Not significantly modulated genes are shown in black. Selected genes of relevant pathways have been labeled. **D** Table with log2(fold change) values of dopaminergic DEGs and statistical significance (SS relative to CTR, *p* adjusted). *Slc6a3*, dopamine transporter; *Slc18a2*, vesicular monoamine transporter member 2; *Ddc*, dopa decarboxylase; *Th*, tyrosine hydroxylase; *Ret*, RET receptor tyrosine kinase; *Aldh1a1*, aldehyde dehydrogenase 1 family member A1; *En2*, engrailed homeobox 2; *Pitx3*, paired like homeodomain 3; *En1*, engrailed homeobox 1; *Drd2*, dopamine receptor D2; *Chrnb3*, neuronal acetylcholine (nicotine) receptor subunit beta-3; *Gucy2c*, peptide guanylate cyclase 2c; *Chrna6*, cholinergic receptor nicotinic alpha 6 subunit; *Gch1*, GTP cyclohydrolase 1; *Ndnf*, neuron-derived neurotrophic factor; *Cplx1*, complexin 1; *Chrna4*, cholinergic receptor nicotinic alpha 4 subunit; *Cplx2*, complexin 2; *Trh*, thyrotropin releasing hormone; *Tac2*, tachykinin 2; *Crhbp*, corticotropin releasing hormone binding protein

To elucidate potential mechanisms involved in the effects of SS on the developing male VTA, we investigated molecular pathways globally modulated by SS by means of RNA-sequencing at the end of the stress procedure (Fig. 4A). Transcriptome analysis was performed by comparing 4 CTR and 4 SS male VTAs at PD 22. SS significantly (*p*adj < 0.05) modulated 81 genes (Supplementary file2): 60 genes resulted downregulated in SS mice, while 21 were upregulated, as depicted in the heatmap (Fig. 4B) and volcano plot (Fig. 4C). Among the downregulated genes, 10 genes are crucial for dopaminergic functionality (Fig. 4D), with 9/10 taking part to the dopaminergic neurogenesis pathway (wikipathways.org/index.php/Pathway:WP1498), encompassing all the phases of neuronal development: the regionalization (engrailed homeobox 1 and 2, *En1* and *En2*), specification (aldehyde dehydrogenase 1 family member A1), differentiation (*En1*; *En2*; paired like homeodomain 3, *Pitx3*) and maturation (tyrosine hydroxylase, *Th*; dopa decarboxylase, *Ddc*; dopamine transporter; vesicular monoamine transporter member 2; *Pitx3*; RET receptor tyrosine kinase). Moreover, some of these genes encode for proteins that are critical for dopamine metabolism, such as the TH and the DDC, and for dopamine activity, such as the DAT and the dopamine receptor D2, DRD2. Other highly significant downregulated genes include the GTP cyclohydrolase 1 gene (*Gch1*), responsible for the production of a TH cofactor, nicotinic cholinergic receptors (cholinergic receptor nicotinic alpha 4 subunit; cholinergic receptor nicotinic alpha 6 subunit; neuronal acetylcholine (nicotine) receptor subunit beta-3), genes associated with synaptic vesicle exocytosis such as complexin 1 and 2 genes, neuron-derived neurotrophic factor, and the peptide guanylate cyclase 2c (*Gucy2c*). Among the few upregulated genes, the most interesting targets were the corticotropin releasing hormone binding protein (*Crhbp*), the thyrotropin releasing hormone (*Trh*), and the neuropeptide neurokinin 2/alpha (tachykinin 2, *Tac2*) (Fig. 4C).

**Fig. 5:**
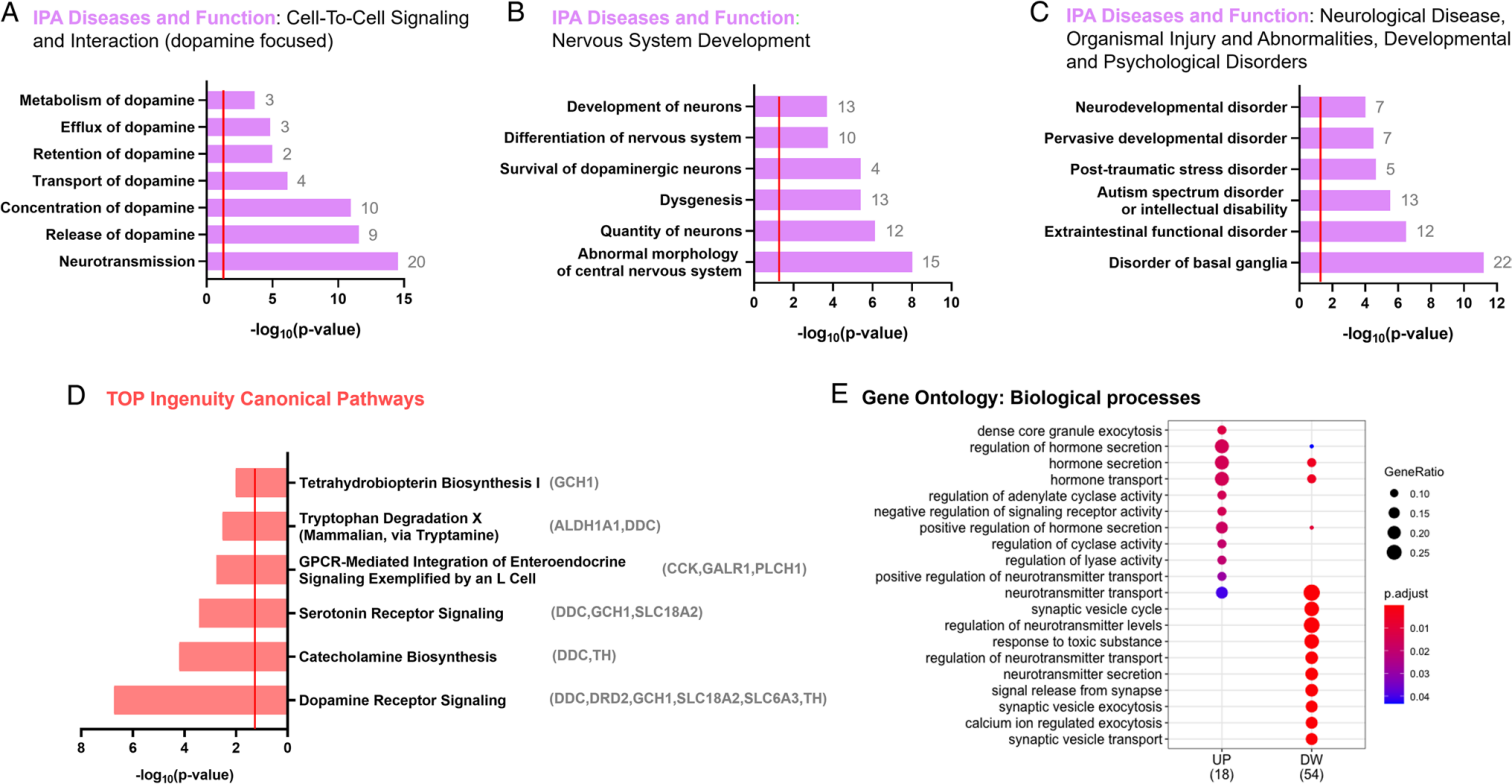
Gene set enrichment analysis of the differentially expressed genes reveals altered developmental trajectories and signaling following SS. **A**–**C** Selected predicted diseases and function annotations affected by SS as revealed by ingenuity pathway analysis (IPA). Annotations are grouped in IPA categories including **A** cell-to-cell signaling and interaction, **B** nervous system development, and **C** neurological disease, organismal injury and abnormalities, developmental and psychological disorders. The number of DEGs relating to each term is provided next to the corresponding bar. **D** Predicted altered ingenuity canonical pathways in SS mice vs CTR with relative DEGs. **E** Dot blot of the TOP 10 enriched up (UP) and downregulated (DW) gene ontology biological processes terms of the DEGs. The size of the dots is based on gene counts enriched in the pathway, while the color shows pathway enrichment significance as indicated in the legend. **A**–**D** The red line indicates *P* = 0.05

Ingenuity pathway analysis (IPA) software was used to determine the functional annotation and to predict the biological pathways affected by SS (*P* < 0.05). We focused on pathways associated with dopaminergic function, developmental processes, and neuro-psychological disorders. Significant diseases or functions annotations have been grouped on the basis of IPA categories in (Fig. 5A–C): Cell-to-cell signaling and interaction (A), nervous system development (B), and neurological disease, organismal injury and abnormalities, developmental, and psychological disorders (C). Based on the pathways predicted, SS broadly affects dopamine functionality, from the metabolism to the release and trafficking, and impairs neurotransmission (Fig. 5A). Moreover, SS influences developmental trajectories, affecting neuronal formation, differentiation, and survival (Fig. 5B), possibly increasing susceptibility to neurological, psychological, and neurodevelopmental disorders (Fig. 5C). TOP predicted altered ingenuity canonical pathways (Fig. 5D) include dopamine and serotonin signaling, catecholamines biosynthesis, GPCR-mediated integration of enteroendocrine signaling exemplified by an L cell, tryptophan degradation X, and tetrahydrobiopterin biosynthesis I. Finally, gene ontology classification revealed alteration in hormonal-related biological processes, exocytosis, and synaptic vesicles trafficking (Fig. 5E).

## Discussion

Stress in early life can disrupt trajectories of dopaminergic system maturation and increase susceptibility to psychopathology and neurological disorders, but the underlying mechanisms are still unknown. In this study, we showed that repeated social stress exposure early in life can alter the development of the DA system at the morphological, molecular, and electrophysiological level, in a sex- and region-dependent manner. Moreover, we showed for the first time an involvement of microglia/immune activity in the mediation of ELS effects on the DA system. Finally, we provided the first transcriptome analysis of the developing VTA immediately after ELS exposure, revealing a series of altered pathways that can be explored in future studies to unveil the biological processes underlying social stress response.

We found that, similarly to other ELS models, SS did not induce reduction in the number [56] and soma size [31] of dopaminergic neurons at PD22. However, we detected changes in the morphology of the dopaminergic cell body. Given that the neuronal soma hosts numerous processes involved in the maintenance of neuron functionality, morphological changes in this compartment may underlie a condition of altered homeostasis induced by the stressful stimulation [33, 57, 58]. Alternatively, since somatic shapes have been used to classify distinct subtypes of neurons [33] in other brain regions (e.g., pyramidal neurons in the cerebral cortex) [59], diverse distributions in shape profiles of DA cells from CTR and SS mice may be due to difference in dopaminergic subpopulations. However, morphological heterogeneity and its functional significance in VTA DA cells have not been characterized yet. In addition to DA cell body changes, SS induced a drastic reduction in the immunoreactivity of TH and DAT, along with decreased DAT currents. These observations confirm and expand literature findings demonstrating that ELS can alter the expression of such targets [11] and may suggest reduced DA synthesis and DAT-mediated responses to extracellular DA in VTA dopaminergic neurons from SS mice. There is growing appreciation of the key role played by the DAT in midbrain regions, which is not only limited to reducing extracellular dopamine levels, but also includes regulation of dopaminergic network connectivity and plasticity [43, 60, 61]. DAT is found in the plasma membrane of the perikaryal, axon terminal, and dendrites of SN and VTA dopamine neurons [55, 62]. Within the midbrain, the extracellular levels of DA contribute to cell excitability and depend on a delicate interplay between DA somatodendritic release and DAT-mediated uptake [63]. Somatodendritic DA release, which is responsible for D2 autoreceptor self-inhibition, involves at least three different mechanisms. First, Ca2 + -dependent vesicular release of DA at low (5–10 nM) or high concentrations (150–400 nM) triggered by spontaneous pacemaker or bursting of VTA and SN DA neurons, respectively [64]. Secondly, other than clearing DA from extracellular space, DAT has been proposed to contribute to somatodendritic DA release via its reverse mode [65, 66]. Finally, in response to low DA concentrations (not enough to activate D2 autoreceptors), DAT-mediated uptake causes an excitatory current which contributes to increase action potential firing [43] and Ca2 + -dependent vesicular release of DA. In this regard, DAT has been considered as ionotropic receptor gated by DA and generating an inward current whose amplitude reflects the degree of its activation [67, 68]. We have previously shown that, at PD22, VTA DA neurons from SS mice displayed reduced self-inhibitory currents induced by exogenous DA application [22]. This reduction, which does not depend on DRD2 functionality [22], may be the result of a complex interaction between altered DAT currents and intracellular and extracellular mechanisms of DA regulation and response. Further studies are necessary to understand the mechanisms underlying these findings and their functional implication on the dopaminergic circuit.

Interestingly, all these SS-induced dopaminergic effects observed at PD22 were present only in males. Current debates in neuroscience highlight the importance of considering “sex as a biological variable” in basic and translational research, particularly in the context of ELS, since male and female brains and bodies mature at different rates, and under different biological and environmental conditions [69, 70]. In humans, certain types of child maltreatment have been associated with different dimensions of psychopathology in men and women, and many psychiatric and neurological disorders show a sex bias in incidence, age of onset, and symptomatology, with increased incidence of neurodevelopmental disorders in boys, and of adolescent-onset mood disorders in girls [71–73]. Animal studies have confirmed sex-specific effects of ELS on neural and behavioral development [73, 74], with male offspring having greater risk of adverse proximal effects, as measured in proximity the of pre-pubertal stress procedure (“within the same developmental epoch as the stress exposure”) [70]. Sex differences in the reward DA system may impact developmental trajectories and responses to stress [75, 76], and recent studies have shown opposite genome-wide signatures of response to ELS between adult female and male VTA [77–79]. Moreover, male and female dopaminergic systems are differentially sculpted by microglia during adolescence [16]. Notably, early postnatal/adolescent stress and early immune activation seem to induce greater neuroimmunological responses in males than in females [80]. In the light of this evidence, it can be hypothesized that the specific male response to SS that we detected may be due to higher sensitivity of the dopaminergic and/or immune system in males compared to females during this developmental window, and/or to sex-different timing in brain responses to stress, which in males may be expressed in proximity to the stress. Further studies are needed to investigate whether SS affects female VTA and/or other brain regions at different time points across the life span.

Consistent with previous literature [11], we detected a regional effect in the dopaminergic responses to SS, with changes being significant in the VTA but not in the SN. Indeed, VTA DA neurons are known to be particularly responsive to “psychological”/behavioral stressors [28, 53, 81], and express higher levels of transcripts related to synaptic plasticity and neuropeptides compared to the SN [82]. The sensitivity of the VTA may be also due to specific neuron-microglia communication, since VTA microglia is prominently different from other basal ganglia, namely nucleus accumbens, cortex and SN microglia [83].

We found that inhibition of SS-induced microglia/immune activation through minocycline prevented some alterations in the morphology of DA neurons and DAT and TH levels in the VTA of male SS mice. Therefore, it is plausible to suggest a role for neuroimmune pathways in the regulation of SS-induced altered development of the DA system. Disentangling neuroimmune pathways is incredibly challenging, and in our setting, it is even more so because minocycline mechanism of action is still not well known. However, minocycline has been extensively used to specifically inhibit microglia activation in response to a variety of different stimuli [26, 84–86], with similar results to more specific compounds (e.g., PLX3397, GW2580) [22, 87]. In microglia, minocycline is known to prevent/reduce stimulus-induced proliferation, production of proinflammatory mediators, phagocytic activity, and morphological and molecular shifts associated with a reactive phenotype [84, 85, 88]. Moreover, it has beneficial effects on dopaminergic neurotoxicity, neuronal apoptotic mechanisms, oxidative stress, glutamate dysfunction, and peripheral inflammation, all processes that influence and are influenced by microglia [84, 89, 90]. Importantly, minocycline effects on microglia and the dopaminergic system may also be the result of inhibited peripheral immune responses, which are known to play a central role in psychosocial stress response [91, 92].

To unravel possible pathways of SS response, we performed RNA-sequencing in the male developing VTA (PD22) at the end of the stress procedure. Our transcriptome analysis confirmed massive downregulation of numerous dopaminergic markers, including TH and DAT genes, and alterations in pathways involved in the synthesis, signaling, and degradation of DA. This effect is not a consequence of reduction in dopaminergic neurons, as CTR and SS mice exhibit the same number of DA cells in the VTA, but it is probably due to transcriptional regulation processes that ultimately affect protein expression, as we demonstrated for TH and DAT. Furthermore, pathway analysis predicted alterations associated with neurodevelopmental trajectories and neurological and psychiatric disorders. Overall, transcriptome, immunohistochemical, and electrophysiological data suggest that SS interferes with developmental programs of the male VTA, possibly inducing diminished DA signaling at the end of the ELS procedure (PD22). Reduction of DA function may be an adaptive response to the repeated, inescapable stress that pups face [93]. It has been shown that DA transmission between the third and fourth postnatal week is critical for the maturation of DA circuitry [94], and developmental DA perturbation has been associated with altered behavioral phenotypes that resemble mental disorders (e.g., attention deficit hyperactivity disorder, substance use disorders [9, 14, 95]). In clinical research, aberrant VTA maturation has been observed in children and adolescent exposed to early life stress or trauma [81, 96].

Microglia and immune pathways may be important regulators of the observed dopaminergic effects, since minocycline was able to rescue the deficits in DAT and TH induced by SS. It has been shown that immune-derived cytokines and prostaglandins cause reductions in DA synthesis, release, and DA metabolites, possibly via depletion of tetrahydrobiopterin (BH4), the cofactor of phenylalanine hydroxylase and TH [97–99]. This neuroimmune pathway has been proposed as the underlying mechanism in inflammation-induced psychiatric symptoms associated with impaired dopamine transmission, such as anhedonia [97, 100, 101]. Interestingly, our RNA-seq revealed alteration of the tetrahydrobiopterin biosynthesis I pathway in SS mice, due to the reduction in the *Gch1* gene, the rate-limiting enzyme in the production of BH4 [102] whose deficiency has been associated with dopaminergic-related diseases, including Parkinson’s disease [103]. Additionally, a highly significant modulation of genes and pathways associated with cholinergic and serotonergic transmission suggests the presence of complex local interactions between different neurotransmitters systems underlying VTA SS response.

Among the few upregulated pathways, we found hormonal and peptide signaling pathways. The related DEGs include markers that are canonically involved in hypothalamic–pituitary–adrenal axis-VTA responses to stress and drugs of abuse, such as *Crhbp* [13], and new targets that merit further consideration. For example, we found strong upregulation of the *Tac2* and *Trh* genes. *Tac2* is upregulated in the brain after chronic social isolation stress [104], and its downstream pathways can affect dopaminergic release from the VTA [105]. *Trh* is involved in the regulation and release of thyroid-stimulating hormone (thyrotropin) and prolactin from the pituitary gland. In the VTA, it is possibly expressed by a sub‐group of GABA and/or glutamate neurons or coming from hypothalamic afferent fibers [106]. TRH seems to be involved in regulating behavioral, energy, and immune homeostasis in the periphery and the brain, including the ventral midbrain, where it exerts a neuroprotective role under oxidative stress conditions in dopaminergic neurons [107]. Moreover, thyroid hormone and associated proteins have recently been implicated in regulation of sex-specific neurodevelopment and response to adolescent stress in animals, and post-traumatic stress disorder in humans [78, 108, 109]. A significantly downregulated neuropeptide gene is *Gucy2c*, which encodes a transmembrane receptor for the intestinal hormone uroguanylin that takes part in the gut-brain endocrine axis regulation of feeding behavior. In the brain, it is selectively expressed by the hypothalamus and dopaminergic neurons from the VTA and SN and distributed in their afferent sites [110]. It is involved in the development of chronic defeat stress-induced depressive behavior and its substrate uroguanylin is decreased peripherally in socially defeated mice [111, 112]. Intriguingly, IPA analysis revealed enrichment in the extraintestinal functional disorder and GPCR-mediated integration of enteroendocrine signaling exemplified by L cell pathways. The genes within our dataset relating to these pathways encode for peptides found in the gut and in the brain and involved in gut-brain communication, neurodevelopment, and behavior [113–116].

In light of these results, we speculate that during social stress exposure, the VTA may be a station where neurotransmitters, hormones, peptides, and immune molecules signal internal homeostatic states from the brain and the periphery to influence central dopaminergic neurotransmission and establish appropriate learning and predictions [98, 117]. This, in turn, would affect future emotional/motivational behavioral choices, which in the case of chronic stress may be maladaptive and increase psychopathological risk [53]. In this setting, microglia would play a central role, since they can sense and read all the signals coming from nervous and peripheral tissues [118]. It can be hypothesized that SS-stimulated microglia mount an inflammatory response that would end up negatively contributing to the observed dopamine neuron changes and DA reduction in SS male VTA. Unfortunately, several technical issues did not allow us to better explore microglia contribution. In particular, the mouse strain employed in this study (DBA/2) made it impossible to take advantage of genetic mouse models of microglia tagging and manipulation, which are based on the C57BL/6 strain. This, in combination with the small size of the developing mouse VTA, meant that we were not able to extract vital microglia cells that could be employed for transcriptomic or in vitro studies. Validation of the SS model on the C57BL/6 strain will prove instrumental to investigations of the specific role of microglia in this setting.

A possible limitation of the study concerns the fact that we stopped analyzing females after we detected significant effects only in males. Including female mice in subsequent experiments (minocycline treatment, RNA-sequencing) would have been advantageous to better explore the observed sex differences and to draw more reliable conclusions on the potential of minocycline to prevent SS-induced dopaminergic aberrations. Future efforts should be made to address this limitation, and to detect which brain substrates are affected by SS in female mice at different developmental time points, in order to provide better knowledge on possible sex-specific pathophysiology in chronic medical conditions.

Overall, these results suggest important targets of developmental repeated social stress effects, with sex and regional specificity, and involvement of microglia and immune system in the mediation of these effects. Additional investigations are needed to evaluate whether the SS-induced developmental dopaminergic alterations described here are maintained in adulthood, or whether different adaptations will occur in the system, and the possible causal relationship with psychopathological risk. A better understanding of such pathways is critical for improving strategies that counteract aberrant brain maturation and psychopathological outcomes following adverse early experiences.

## Supplementary Information

Below is the link to the electronic supplementary material.

Supplementary file1 (PDF 192 KB)

Supplementary file2 (CSV 10 KB)

## Data Availability

All source data supporting the findings of this manuscript are available from first and corresponding authors upon request. RNA-Seq data are available through NCBI’s Gene Expression Omnibus (GEO) repository, under accession number GSE199136.
