# Supplementary material for: Early Life Social Stress Causes Sex- and Region-Dependent Dopaminergic Changes that Are Prevented by Minocycline
Source: Mol Neurobiol. 2022 Apr 18;59(6):3913–32. doi: 10.1007/s12035-022-02830-6 (PMC9148283; doi:10.1007/s12035-022-02830-6)
Supplement: Supplementary file 1 — Supplementary file1 (PDF 192 KB) [file 12035_2022_2830_MOESM1_ESM.pdf]

Early life social stress causes sex- and region-dependent dopaminergic changes that are prevented by minocycline

Clarissa Catale[1], Luisa Lo Iacono[2], Alessandro Martini[3], Constantin Heil[4], Ezia Guatteo[5], Nicola Biagio Mercuri[6], Maria Teresa Viscomi[7], Daniela Palacios[8#], and Valeria Carola[9*#]

Affiliation

1 Division of Experimental Neuroscience, Neurobiology of Behavior Laboratory, IRCCS Santa Lucia Foundation, Rome, Italy.

2 Department of Dynamic and Clinical Psychology, and Health Studies, Sapienza University of Rome, Rome, Italy.

3 Division of Experimental Neuroscience, Experimental Neurology Laboratory, IRCCS Santa Lucia Foundation, Rome, Italy.

4 Division of Experimental Neuroscience, Epigenetics and Signal transduction Laboratory, IRCCS Santa Lucia Foundation, Rome, Italy.

5 Department of Motor Science and Wellness, University of Naples Parthenope, Naples, Italy; Division of Experimental Neuroscience, Experimental Neurology Laboratory, IRCCS Santa Lucia Foundation, Rome, Italy.

6 Department of Systems Medicine, Università degli Studi di Roma Tor Vergata, Rome, Italy; Division of Experimental Neuroscience, Experimental Neurology Laboratory, IRCCS Santa Lucia Foundation, Rome, Italy.

7 Department of Life Science and Public Health, Section of Histology and Embryology, Università Cattolica Del S. Cuore, Rome, Italy; IRCCS Fondazione Policlinico Universitario A. Gemelli Rome, Italy.

8 Department of Life Science and Public Health, Section of Biology, Università Cattolica Del S. Cuore, Rome, Italy; IRCCS Fondazione Policlinico Universitario A. Gemelli Rome, Italy.

Previous address: Division of Experimental Neuroscience, Epigenetics and Signal transduction Laboratory, IRCCS Santa Lucia Foundation, Rome, Italy.

9 Department of Dynamic and Clinical Psychology, and Health Studies, Sapienza University of Rome, Rome, Italy; Division of Experimental Neuroscience, Neurobiology of Behavior Laboratory, IRCCS Santa Lucia Foundation, Rome, Italy.

Equal senior author

* Corresponding author e-mail: valeria.carola@uniroma1.it.

Supplementary information

Supplementary figures

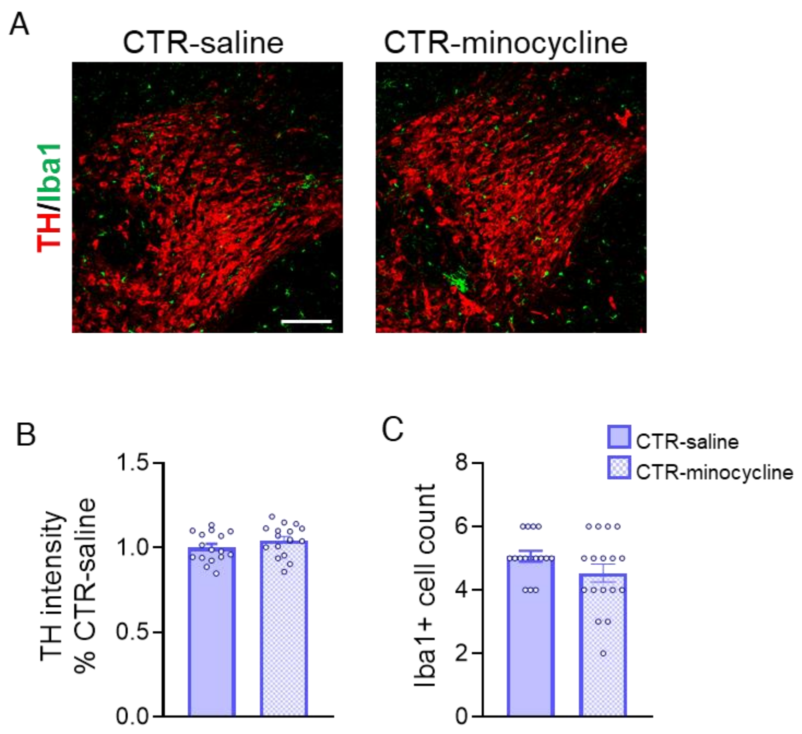

Supplementary fig. 1. Developmental minocycline exposure does not alter the expression of TH nor the number of microglia cells in the VTA at PD22. (A) Representative single hemisphere confocal images of TH and Iba1 (microglia) immunoreactivity in the VTA of PD22 pups injected

with either saline or minocycline from PD14 to 21. **(B, C)** Quantification of TH intensity and microglia cell number revealed no significant differences between CTR-saline and CTR-minocycline mice (N= 3 animals/group, 5-6 sections/animal). Results are presented as mean±sem, dots in the graphs represent sections. (A) Scale bar = 100 µm.

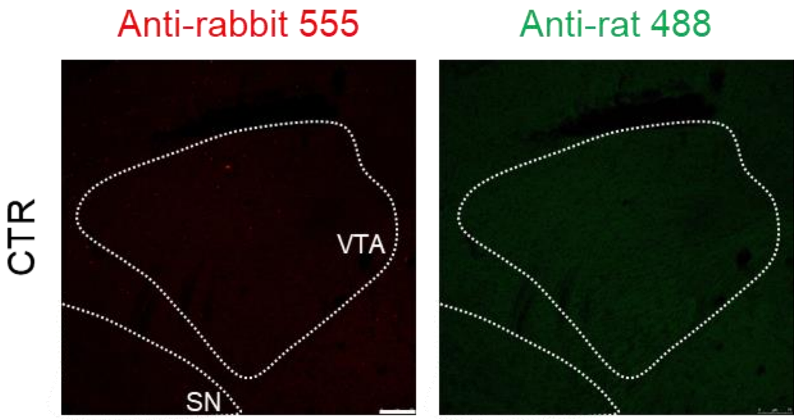

Supplementary fig. 2. Antibodies specificity controls. To evaluate specificity of immunofluorescence labeling, coronal sections of midbrain were incubated with normal serum in the absence of primary antibody and then with the same secondary antibodies used for experiments, including Alexa Fluor 555 conjugated donkey anti-rabbit (left) and Alexa Fluor 488 conjugated donkey anti-rat (right). No non-specific staining was detected in either the VTA or Substantia Nigra (SN).

Supplementary table legend

Supplementary Table 1. List of differentially expressed genes. Differentially expressed genes were obtained by DESeq2 using default parameters (p-adjusted <0.05). For each gene, the table shows the base mean in CTR samples (baseMean), the log2 Fold change in SS mice (log2FoldChange), the

standard error of the log2FoldChange (lfcSE), the test statistics (stat), the p-value (pvalue), and the corrected p-value (padj).

Supplementary methods

Minocycline treatment

CTR pups received daily intraperitoneal (i.p.) injection of minocycline hydrochloride (50 mg/kg; Sigma-Aldrich, #M9511) dissolved in sterile saline solution (2.5 mg/ml) or sterile saline solution from PD14 to PD21.

Histological procedures

To measure Iba1 and TH expression, 3 male mice/group were sacrificed at PD22 as described in the main text. Processing of the brains and immunofluorescence were performed as described in the main text. The primary antibody cocktail included mouse anti-TH (#MAB318, Merck Millipore) and rabbit anti-Iba1 (#019-19741, Wako Lab Chemicals) to detect microglia. The secondary antibodies cocktail included Alexa Fluor 555 conjugated donkey anti-rabbit (#A-31572, 1:200; ThermoFisher Scientific) and Alexa Fluor 488 conjugated donkey anti-mouse (#A-21202). Acquisitions were carried out on a confocal laser scanning microscope (Zeiss CLSM800) through the 20x/0.50 objective (Plan-Apochromat, Zeiss).

Quantitative analysis of fluorescence images

Analyses were performed by using the ImageJ software (31). TH intensity was quantified as described in the main text. For quantification of microglia in the VTA, all labeled Iba1+ cells within a squared box (VTA: 200 μm per side) randomly positioned in 5-6 regularly spaced sections per animal were counted. Only immunolabeled cells with a distinct nucleus (DAPI-positive) in the focal plane were considered.

Statistics

Data were first checked for normality by graphical inspection of the residuals' distribution and then analyzed by parametric Student's *t*-test (*t*-test). Significance was set at $P < .05$. Statistica software Version 12.0 (StatSoft, Tulsa, OK, USA) was used to perform the statistical analyses.
